# Perinatal Polyunsaturated Fatty Acid Status and Obesity Risk

**DOI:** 10.3390/nu13113882

**Published:** 2021-10-29

**Authors:** Hans Demmelmair, Berthold Koletzko

**Affiliations:** Division of Metabolic and Nutritional Medicine, Department Pediatrics, Dr. von Hauner Children’s Hospital, University of Munich Medical Centre, LMU—Ludwig-Maximilians-Universität Munich, D-80337 Munich, Germany; hans.demmelmair@med.uni-muenchen.de

**Keywords:** polyunsaturated fatty acid, obesity, perinatal period, arachidonic acid, eicosapentaenoic acid, docosahexaenoic acid

## Abstract

High obesity rates in almost all regions of the world prompt an urgent need for effective obesity prevention. Very good scientific evidence from cell culture and rodent studies show that the availability of essential polyunsaturated fatty acids (PUFA) and their long-chain polyunsaturated derivatives, namely, arachidonic acid, eicosapentaenoic acid and docosahexaenoic acid, influence adipogenesis; for this reason, early life status may influence later obesity risk. The respective PUFA effects could be mediated via their eicosanoid derivatives, their influence on cell membrane properties, the browning of white adipose tissue, changes to the offspring gut microbiome, their influence on developing regulatory circuits, and gene expression during critical periods. Randomized clinical trials and observational studies show divergent findings in humans, with mostly null findings but also the positive and negative effects of an increased n-3 to n-6 PUFA ratio on BMI and fat mass development. Hence, animal study findings cannot be directly extrapolated to humans. Even though the mechanistic data basis for the effects of n-3 PUFA on obesity risk appears promising, no recommendations for humans can be derived at present.

## 1. Introduction

The influence of dietary fatty acid (FA) groups and individual FAs on human health has long been recognized [1]. In the perinatal period, the long-term effects of early life events also come into play; this is referred to as the developmental origins of long-term health and disease [2,3]. Early nutrition or metabolic programming assumes that nutritional experience during critical periods in early life, both pre- and post-natal, can program the developmental trajectories of individuals with respect to metabolism and health [4]. This has been clearly shown in animal models, and ample data from retrospective observational studies suggest the existence of their corresponding effects in humans [5].

**Figure 1:**
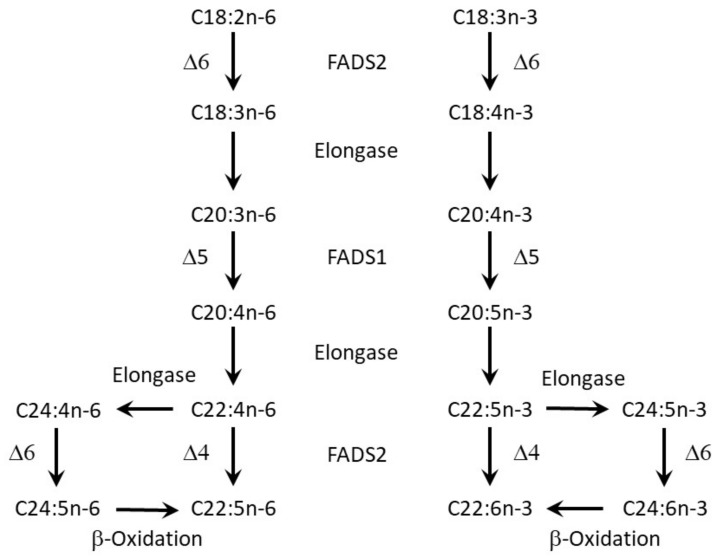
Synthesis of n-3 and n-6 long-chain polyunsaturated fatty acids (LC-PUFA) in mammals from C18:3n-3 (α-linolenic acid) and C18:2n-6 (linoleic acid), as described by Zhang et al. [15].

Among FAs, polyunsaturated FAs (PUFA) are important since they cannot be built up de novo by the human organism. From the essential FA linoleic acid (18:2n-6, LA), the n-6 series LA derivatives dihomo-γ-linolenic acid (C20:3n-6) and arachidonic acid (C20:4n-6, ARA) are derived. The precursor n-3 PUFA is the essential α-linolenic acid (C18:3n-3, ALA), and the main ALA derivatives are eicosapentaenoic acid (C20:5n-3, EPA) and docosahexaenoic acid (C22:6n-3, DHA). Humans can synthesize the derivatives of the n-3 and n-6 series from essential FAs via desaturases and elongases (Figure 1) but the interconversion of n-3 and n-6 PUFA is not possible [6]. However, the capacity for endogenous conversion of essential FAs to long-chain polyunsaturated fatty acids (LC-PUFA) is rather limited in humans. For the n-6 series, endogenous conversion seems of higher importance for LC-PUFA levels than for the n-3 side, due to the higher intake of LA compared to ALA [7]. The endogenous synthesis is of relevance for the DHA level in individuals with a low intake of LC-PUFA [7]. ALA intake can not only increase EPA levels but also DHA levels. This has been shown in infants and in women, where estrogen seems to increase conversion activity [8,9,10,11]. Nevertheless, dietary intake from fish or via supplements is the most important determinant of n-3 LC-PUFA status [12,13]. This agrees with the observation that genotypes of the desaturases strongly influence the levels of n-6 LC-PUFA, while they are of much less relevance for n-3 LC-PUFA [14].

Interest in the health consequences of n-3 LC-PUFA status has been stimulated by the increase in the dietary intake of n-6 FAs and the corresponding decrease in n-3 FAs during the 20th century, which has been associated with the concomitant increase of obesity and of allergic manifestations during the same time period [16,17].

Genetic and dietary influences on PUFA status apply to humans of any age and, thus, contribute to the FA status during the perinatal period. Support for the importance of the relative LC-PUFA contribution to total FAs for infant development comes from the observation that LC-PUFA are enriched in the fetal stores by preferential transfer from the mother to the fetus via the placenta during pregnancy [18,19]. While percentages of saturated FAs are similar, LA and ALA have significantly smaller percentages in cord blood phospholipids than in maternal phospholipids [20]. In contrast, LC-PUFA contribute far higher percentages to cord blood than maternal blood lipids [20]. While the concentration-driven transfer of non-esterified FAs after the hydrolysis of lipids before uptake or during placental metabolization contributes to placental FA transfer, the preferential transport of ARA and DHA relative to other FAs is facilitated by specific binding and transfer proteins [21]. The major facilitator superfamily domain-containing protein 2A (MFSD2A), which is known for the transfer of DHA containing lysophospholipids across the blood-brain barrier, appears to also facilitate DHA transfer across the placenta to the fetus [22].

After delivery, human milk or infant formula provides FAs to the infant. This is associated with a decrease in the percentage of ARA and DHA in infant serum phospholipids, while LA and ALA increase [23]. LC-PUFA can be produced in limited amounts by endogenous synthesis from essential FAs in babies, but this does not balance out the absence of dietary intake [24]. This decrease is observed although human milk globally provides on average 0.3% of fat as DHA (range: 0.1–1.4%) and 0.5% ARA (range: 0.2–1.0%), based on 65 studies from different countries [25]. This intake leads to higher plasma LC-PUFA concentrations in infants fed human milk rather than formula without LC-PUFA [26]. Most of the fat in infant formula is provided by plant oil supplying essential FAs. Aiming at similar plasma LC-PUFA concentrations and related biological benefits in human milk- and formula-fed infants, infant formulas that are in use nowadays contain DHA from different sources [13], and concomitant enrichment with ARA is recommended [27].

The relationships between the dietary LC-PUFA intake of mothers and infants and their FA status are well established, and research now aims to describe the associations between perinatal PUFA status and the short- and long-term development and health risks of the infant. The major areas of research are cognitive function, atopic diseases, and the long-term risks of obesity and related diseases [28].

Childhood obesity is a critical health issue, as it tends to persist into adulthood [29] and markedly increases the risk of chronic diseases in later life, including metabolic disorders, insulin resistance and diabetes, cardiovascular diseases, non-alcoholic steatohepatitis, some forms of cancer, musculoskeletal, and psychological disorders and much higher mortality, which makes it an important health-risk factor [30]. The importance of this issue becomes clear from the high number of affected children. For the period of 2015–2017, it was estimated that in Europe, 9–43% of the boys and 5–43% of the girls in the age range of 6–9 years were overweight; 2–21% of the boys and 1–19% of the girls, respectively, were obese [31].

In most cases, obesity results from the inability of the individual to adapt his/her behavior to obesogenic pressure from the environment [32]. Personal factors, including genetic and environmental factors, such as food availability, culture, area of residency, caregiver behavior, and sports facilities’ availability, affect the risk of obesity [32].

Current treatment options for obesity in adults and children are less than satisfactory, often costly, and with only a modest duration of results [33]. Therefore, prevention of obesity needs to be prioritized, with the establishment of behavioral changes from early life onward, including better dietary and physical activity behaviors. The aim of this narrative review is to report on potential opportunities for influencing anthropometric development and associated obesity risk through modifying the perinatal PUFA and LC-PUFA status.

## 2. Experimental Models

Maternal obesity at the onset of pregnancy, and high weight gain during pregnancy, increase the risk of later obesity in the offspring [34,35]. Scientific interest in this question has been raised by proposed mechanisms that potentially mediate the effects of perinatal PUFA status on body-fat accumulation in the offspring. The perinatal FA status corresponds to maternal FA levels during pregnancy. Fetal FA status is usually determined via cord blood analyses, and infant FA status relates to blood FA levels during the breast- or formula-feeding period. Mechanistically, there is primarily the role of PUFA via LC-PUFA as eicosanoid precursors, but there is also the influence of FAs on cell membrane composition, the browning of white adipose tissue, the offspring’s gut microbiome, the development of regulatory circuits, and gene expression during this perinatal period.

### 2.1. Fatty Acids and Eicosanoids

Ailhaud and colleagues incubated preadipocytes with ARA and found an increased growth and differentiation of adipocytes [36]. They reported that the adipogenic effect of ARA could partially be blocked by cyclooxygenase inhibitors but could also be induced by analogs of PGI2 (prostacyclin). Thus, ARA may be converted to the eicosanoid PGI2, which interacts with the cell surface prostacyclin receptor IP. Through the IP/prostacyclin system, the protein kinase A pathway is activated to induce adipocyte differentiation and growth. In contrast, EPA and DHA, from which prostacyclin is not derived, barely induce adipocyte differentiation [37] and could even suppress the storage of lipids in adipocytes [38]. All long-chain FAs act directly—without further metabolization—as transcriptional regulators of peroxisome proliferator-activated receptors (PPARs) [39]. Interestingly, ARA and some of its metabolites derived from cyclooxygenase and lipoxygenase activities are also activators of PPARs. This indicates the greater importance of ARA, relative to saturated, monounsaturated and n-3 LC-PUFA, regarding the early and later phases of adipogenesis [39].

Feeding female mice before mating and during the pregnancy/suckling period with a diet high in fat and in the omega-6/3 ratio, with an LA to ALA ratio of 59 to 1, led to higher body weight, fat mass, epididymial fat pad weight and adipocyte size in the offspring than when feeding an isocaloric diet with a LA to ALA ratio of 2 to 1 [40]. The pups of the mothers on the 59 to 1 diet were 50% heavier at weaning than those fed the low-LA diet. During this early period of life, adipose tissue is extensively deposited, and this difference in body weight remained until adulthood. The introduction of ALA into the diet prevented enhanced adipogenesis, which is in line with the adipogenic effect of n-6 PUFA as observed in vitro. The importance of the LA-derived ARA and the ARA-derived prostacyclin is supported by comparing this observation in wild-type mice to the results of the same experiment in prostacyclin receptor knock-out mice [40]. Contrary to the wild-type mice, prostacyclin receptor-null mice show no higher body weight and fat mass of pups from mothers fed the high-LA diet, compared to the lower-LA or standard diet. This shows that the ARA-derived PGI2 signaling is mandatory for the increase of the fat mass of pups on an LA-enriched diet [40].

The mice study is in line with the observation that the addition of ARA can increase PGI2 and, correspondingly, the differentiation of preadipocytes to adipocytes in cell cultures [36], although the effect may depend on further factors [41]. The increase in PGI2 in response to high ARA levels was confirmed in the adipose tissue of guinea pigs supplemented with ARA from post-natal day 5 to day 21 [42]. Nevertheless, this was not accompanied by a higher fat mass in the supplemented group, although PGI2 levels were higher until day 105. This could be a difference between species, as the intensive growth of fat depots in guinea pigs occurs earlier than in mice. This points toward the possible existence of critical early time windows for the adipogenic effect of ARA and also emphasizes that findings in animal models may not necessarily apply to humans.

Herring- and beef-based diets fed during gestation and lactation to C57BL/6 mice yielded a clearly higher content of n-3 LC-PUFA in the herring-fed animals in milk and several offspring tissues [43]. After weaning, half of the pups in each group were switched to the other diet. At 9 weeks of age, the offspring of herring-fed dams had less body fat than the offspring of beef-fed dams, which did not depend on the diet after weaning. Reduced body fat in the offspring was only observed when the maternal diet included n-3 LC-PUFA. However, the postnatal diet had a significant influence on insulin sensitivity, plasma lipids and the amount of brown adipose tissue.

Feeding the male offspring of C57Bl/6 mice a diet with plant oils only or with 80% vegetable oils plus 20% fish oil (as the source of n-3 LC-PUFA) from days 2 to 42 of life, followed by an n-6 FA-rich diet without LC-PUFA until day 98, did not change the body weights but the n-3 LC-PUFA-rich diet reduced fat accumulation by ~30% and reduced hypertrophic adipocytes at day 98; it also improved the plasma lipid profiles [44]. Apparently, relatively moderate alterations of fat quality during early postnatal life can markedly affect later metabolic health.

Wielinga and colleagues fed an ARA/DHA or an EPA/DHA mixture to ApoE*3-Leiden transgenic mice, an animal model with blood lipids more similar to humans, from postnatal weeks 4 to 12, followed by a high fat and high carbohydrate diet without LC-PUFA until 20 weeks of age [45]. ARA/DHA-fed mice gained less bodyweight in comparison to controls, which received the same chow during the whole study until weeks 12 and 20. EPA/DHA had no significant effect on weight compared to the control diet. At 20 weeks, the trend regarding fat mass was similar, and both supplements tended to reduce adipocyte cell size and cholesterol and triglyceride in the circulation. This confirms the potential of LC-PUFA to affect body weight and lipid metabolism beyond the supplementation period. Furthermore, the study shows the complexity of the influence of LC-PUFA on weight development, as certain combinations of ARA and DHA may be more effective than purely increasing n-3 LC-PUFA.

Further complexities of the effects of LC-PUFA during the perinatal period on offspring development can be seen in a more recent experiment with Wistar rats. Four groups, of 6–9 female rats each, were fed diets containing different fat levels (18% or 36% on a weight basis) and with different LA to ALA ratios (9:1 or 1:1.5) during pregnancy and lactation, while their offspring were weaned to standard chow at 3 weeks of age [46]. Only those offspring on a low-LA/low-fat diet had significantly higher percentages of blood n-3 LC-PUFA and higher gonadal fat mass at 4 weeks of age. Independent of the FA composition, male and female offspring of high-fat diet-fed dams were about 20% lighter than the offspring of the low-fat diet-fed dams, and this difference continued to be significant until the age of 8 weeks [47]. The study also showed lower gonadal fat (as a percentage of body weight) in the low-fat and low-n-6 group at the age of 4 weeks but not at 8 weeks. Sex-specific interactions of fat content and FA composition were observed for the RNA expression of FA synthase, PPAR gamma, and leptin [47]. Thus, while offspring fat deposition seemed sensitive to both the maternal dietary LA to ALA ratio and the total fat content, offspring growth and the lipogenic capacity of tissues seemed more sensitive to total fat in the maternal diet. This study confirms that metabolic programming effects are often reported as sexually dimorphic [48]. It is very conceivable that even a temporary change in gene expression may have long-term consequences. An important finding of the study is that other factors besides dietary FA composition might be of more relevance to the obesity risk. This is outlined in the findings for the fat content of the diet, which by itself also might influence offspring development, and it must be taken into account that the effects of any dietary FAs might be attenuated if such FAs are diluted in a huge amount of other fat. A further point of potential relevance is the limitations of the endogenous conversion of the dietary essential FAs to their corresponding bioactive LC-PUFA derivatives, which is usually quite effective in rodents but is influenced by the competition of LA and ALA, respectively, for enzymatic conversion, whereas conversion might be further limited by a low protein intake from isocaloric diets with a high fat content [47,49].

### 2.2. Fatty Acids and Microbiome

A further mediation possibility of the influence of perinatal LC-PUFA status on obesity risk has been indicated in studies using the fat-1 transgenic mouse model [50]. Fat-1 transgenic mice are genetically engineered to convert n-6 PUFA into n-3 PUFA, which enables studies with identical diets but different PUFA levels in the animals [51]. Offspring of wild-type and fat-1-mother mice were studied. N-3 PUFA and n-3 LC-PUFA were significantly higher in all offspring, which were either born from fat-1 or cross-fostered to fat-1 dams; in the offspring, a lasting effect on the gut microbiome, with higher Epsilonproteobacteria, Bacteroides, and Akkermansia and a lower relative abundance of Clostridia was also observed [50]. In agreement with other studies, lasting higher body weight was only observed in male mice. The offspring mice were fed the same high-fat diet after weaning. While the FA status was no longer different between the groups, differences in the microbiome and in the bodyweight of males persisted, which suggested that the microbiome might be involved in mediating the effect of FAs on obesity risk [50].

### 2.3. Fatty Acids and Appetite Regulation

Rodent studies also point toward the influence of perinatal LC-PUFA status on postnatal leptin levels and the establishment of hypothalamic satiety-regulatory circuitry, which includes the expression of hypothalamic appetite regulators, such as neuropeptide Y (NPY), pro-opiomelanocortin (POMC), and agouti-related protein during the early postnatal period [52,53]. Schipper et al. fed mice early post-natal diets that were different from the n-3/n-6 ratio, which affected the density of agouti-related protein and alpha-melanocorticoin immunoreactive fibers in the paraventricular nucleus at day 28 [52]. Although the diets did not induce weight differences, the differences in the neural projections to the paraventricular nucleus of the hypothalamus persisted into adulthood and were still apparent at postnatal day 98, indicating that perinatal PUFA intake may induce lasting effects on energy regulation. Xavier et al. showed in rats that a high n-3 diet fed to dams during pregnancy leads in offspring to lower weight according to length, reduced circulating leptin, and sex-specifically affects the expression of neuropeptides in the hypothalamus [53]. It must be noted that not all studies have confirmed corresponding effects on weight development, and long-term effects have not consistently been demonstrated. However, a common pattern is that LC-PUFA effects are sexually dimorphic and that there are influences by macronutrient composition [47]. Investigations in rodents also tested the effects of the perinatal n-3/n-6 ratio in the diet and correspondingly in brain tissue on offspring food preferences. Whether perinatal n-3 dietary deficiency influences the desire for flavorsome food, interacts with a later high-fat diet and increases body weight in the offspring is not yet clear, but this has been studied in pregnant C57Bl6 mice [54]. Dams and pups were subjected to n-3 adequate (3.0 g algal DHA + 4.8 g flaxseed oil per kg of food) or n-3 deficient diets without DHA and flaxseed oil until postnatal day 21 and were then weaned to a standard chow or high-fat diet. Adult male offspring were subjected to a progressive ratio operant task for sucrose-motivated behavior or were granted longer feeding periods with a saturated high-fat diet or chow, followed by the determination of energy balance and anxiety-like behavior in the elevated plus maze and open field test. Dietary n-3 deficiency lowered brain n-3 levels, with a corresponding decrease in the n-3/n-6 ratio from 2.1 to 1.3, augmented the rewarding effects of sucrose, increased weight and fat accumulation, lowered physical activity, and increased anxiety-like behavior independent of diet in the open field. On the other hand, the behavior of the animals in the elevated plus maze was not influenced by perinatal n-3 availability but instead by a later high-fat or standard chow diet [54]. Thus, early life dietary n-3 deficiency can facilitate reward-motivated behavior and decrease physical activity, which might, together with other obesogenic effects, lead to obesity.

The potential long-lasting effects of n-3 deficiency were also shown in a mouse study by Sakayori et al., who report that a diet with a very high n-3/n-6 ratio of 1/120 fed at least during pregnancy upregulates the midbrain dopaminergic system and increases the intake of palatable foods, via higher mesolimbic dopamine release and dopamine receptor expression in neurons [55]. In this elegant experiment, the authors show that in n-6 mice, the hedonic behavior led to the higher consumption of sucrose solutions at the expense of water consumption, compared to n-3 mice. These findings suggest that the maternal consumption of PUFAs can have long-lasting effects on the offspring’s food preferences, which, in combination with the availability of energy-dense palatable food, may induce obesity. As a limitation of their study, the authors point out that in human diets, ratios of 1 to 4–20 are more common than the n-3 to n-6 ratio of 1 to 120 used in their study [56], but the effects of the dietary n-3/n-6 ratio on brain FA composition have also been shown in humans and the consequences for brain development are discussed [57].

### 2.4. Fatty Acids and Brown Adipose Tissue

While the majority of adipose tissue in mammals is energy-storing white adipose tissue, there is also some energy-dissipating brown adipose tissue. Supporting the differentiation of preadipocytes into more brown, mitochondria-rich and uncoupling protein 1 (UCP1)-expressing adipocytes could be a strategy for prevention and management of obesity [58]. It has been found that that the G-protein coupled receptor 120 is probably of importance for mediating the effect on browning; among the agents influencing the browning of adipocytes is the n-3 FA EPA, while DHA seems to have hardly any effect [58]. Of interest for the long-term effects of the perinatal period is a study by Fan et al., who fed a diet to CB57BL/6 mice during pregnancy and lactation that had 15% of calories from palm oil or fish oil [59]. At the time of weaning, body weight and white adipose tissue accumulation were lower in the fish oil-fed offspring. Furthermore, in brown adipose tissue, the n-3/n-6 ratio was clearly higher and the expression of the brown signature genes UCP1, G-protein coupled receptor 120, and PPAR gamma coactivator 1-alpha, cell death activator CIDE-A, and histone-lysine N-methyltransferase PRDM16 was higher. In addition, the expression of several micro-RNAs promoting the browning of adipose tissue was higher in the fish-oil group. In cell culture, histone modifications indicating epigenetic mechanisms to mediate the effects of FAs on browning were shown. After weaning, both groups received diets without n-3 PUFA. At the age of 11 weeks, cold exposure induced significantly higher energy dissipation and higher expression of UCP1 and PPAR gamma coactivator 1-alpha in brown and white adipose tissue in the fish-oil group. Thus, maternal n-3 LC-PUFA supplementation supports fetal brown adipose tissue activation, with apparent long-lasting effects in the offspring. Observations in mice and in cultures of multipotent adipose stem cells suggest not only that EPA positively affects brown adipose tissues but also that the ratio of n-3 to n-6 FAs is of importance, given that the ARA-derived prostaglandins E2 and F2α prevent the conversion of white adipose tissue into brown adipose tissue [60].

Although it seems plausible that the perinatal n-3/n-6 ratio could influence long-term obesity risk, not all mouse studies support the concept that dietary EPA enhances the recruitment of brown or brite adipocytes, but this might be conditional to cold stimulation. Maurer et al. could not induce differences in the weight of white or brown adipose tissue and the expression of UCP1 and cell-death activator CIDE-A after feeding a fish-oil-enriched diet for 3 weeks to adult mice kept under thermo-neutral conditions [61].

**Figure 2:**
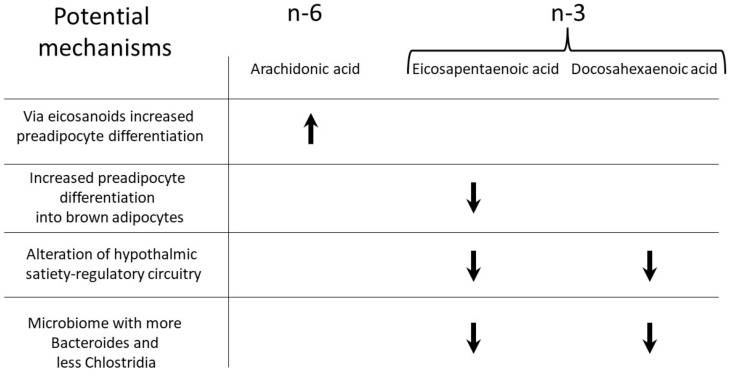
Outline of some of the mechanisms described in animal studies regarding how individual LC-PUFA increase (↑) or decrease (↓) the obesity risk during the perinatal period.

Cell culture and animal studies suggest a variety of very plausible mechanisms for antiobesogenic effects by n-3 FAs in the perinatal period (Figure 2). The studies mainly confirm the beneficial effects of n-3 LC-PUFA. Moreover, there are consistent indications that n-6 LC-PUFA can support adipocyte differentiation and adipose tissue development.

## 3. Human Studies

Findings in humans so far do not consistently support the concept that perinatal n-3 LC-PUFA supply influences infant anthropometric development and decreases the later risk of obesity, as was studied in observational and interventional studies.

### 3.1. Observational Studies

Findings in observational studies, which relate n-3 and n-6 LC-PUFA status to later obesity, are not consistent. In a birth cohort in the US (Project Viva), n-3 LC-PUFA supply during pregnancy, estimated via dietary intake records and the analysis of plasma phosphatidylcholine composition, was negatively associated with adiposity measures at the age of 3 years, while n-6 LC-PUFA showed no obvious effect [62]. In contrast, the Southampton women’s study found n-6 LC-PUFA levels in plasma samples at 34 weeks of gestation that were positively associated with body fat in the offspring at four and six years of age, whereas no influence of n-3 was found [63]. Potentially, the generally lower n-3 to n-6 ratio in the US diet, which may determine the effects of either series of PUFA on obesity, might be related to the different results seen in the US and UK cohorts. Recently, a further evaluation of dietary intake data collected in Project Viva applied a dietary inflammatory index and a Mediterranean diet score, which are both partly determined by the intake of PUFA and antioxidants [64]. In the examined 1459 mother–child dyads, the children of women in the highest dietary inflammatory index quartile had higher BMI-z score growth rates and, at the age of 7–10 years, higher BMI z-scores than the children of mothers in the lowest quartile. Furthermore, the children of mothers in the lowest quartile for the Mediterranean diet score had significantly higher BMI-z-scores than children of mothers from the highest quartile [64]. This could indicate a complex relationship between diet and adipose tissue deposition that is not fully described solely by PUFA intake or blood levels, and the effects of LC-PUFA occur only together with the intake of other nutrients. Long-term dietary habits, which also strongly affect LC-PUFA status and body stores [65], could be at least as important as the current diet during the later phases of pregnancy. This could apply, for example, to the early phase of pregnancy and the pre-conception period, which are not included in most intervention studies that usually only start in mid-pregnancy.

The Generation R study had enrolled pregnant women from 2002 to 2006 and, for 4830 mother–infant pairs, glycerophospholipid FA composition at mid-pregnancy and anthropometric follow-up data of the children at the age of 6 years were available [66]. ALA and the n-3 LC-PUFA were negatively associated with total body-fat percentage and the android:gynoid fat mass ratio, but there were no associations with BMI. Higher n-6-series FAs were related to higher total body fat percentage, higher android:gynoid fat mass ratios, and higher abdominal preperitoneal fat mass area; however, for the n-3 FAs, no significant association with BMI was observed. This speaks for the influence of FAs during pregnancy on long-term adipose tissue development and also emphasizes the importance of detailed anthropometric examinations, as BMI variance in the children was fully explained by maternal BMI and further confounding factors, and is known to be of limited value in describing adiposity [66,67]. In the Generation R cohort, the positive association between n-3/n-6 ratio and birth weight and pregnancy duration could also be confirmed [68]; specifically, DHA was positively associated with higher total and HDL cholesterol at the age of 6 years [69].

An observational study in Copenhagen measured human milk FA composition at the age of one month and followed 222 children up to the age of 7 years with annual anthropometric measurements [70]. Their BMI until the age of 7 years and fat as a percentage of body weight from the age of 6 to 9 years seemed inversely associated with DHA percentages in the milk samples in boys and girls, although statistical significance was only observed for females [70]. This is somewhat against the findings of an intervention study in Denmark, where lactating mothers with a low habitual fish intake were randomized to take fish oil or olive oil supplements for the first 4 months of lactation. Supplementation induced significantly higher milk fat DHA percentages at four months of lactation (0.4 ± 0.2% vs. 1.3 ± 0.7%) and also a higher BMI and waist circumference at the age of 2.5 years [71,72]. The effect of DHA on BMI could no longer be shown by the age of 7 years, but the potential for long-term effects of early DHA intake was indicated by lower height and higher blood pressure (boys only) in the children from the fish-oil group at the age of 13 years [73,74]. Overall, while observational studies report different associations between maternal PUFA intake or status and offspring obesity, no consistent effects of perinatal LC-PUFA status on later obesity risk can be deduced, as also reported in the meta-analyses of observational studies [75,76].

### 3.2. Randomized Clinical Trials

A prominent example is the open-label randomized INFAT intervention study (impact of nutritional fatty acids during pregnancy and lactation on early human adipose tissue development) that enrolled 208 pregnant women [77]. The study compared dietary supplementation with n-3 LC-PUFA (1020 mg/d DHA plus 180 mg/d EPA), combined with a dietary recommendation to reduce ARA intake from week 15 of gestation to 16 weeks post-delivery, to standard dietary counseling [77]. Analysis of FAs in red blood cell phospholipids, obtained pre- and post-delivery from the mother, cord blood phospholipids, and milk showed that supplementation and a 20% lower ARA intake in the intervention group was effective in increasing DHA and EPA and lowering ARA percentages in blood [78]. There were also effects on n-3 LC-PUFA in milk, with DHA percentages in the intervention group about 4 times higher at both time points of measurement (6 weeks and 16 weeks post-partum), but milk ARA did not differ, with values around 0.4% in both groups at both time points [79]. In infant red blood cells, there were tendencies toward lower ARA and higher DHA levels in the n-3 group but only the ratio differed significantly [78]. The offspring follow-up until the age of five years, with very detailed phenotyping, is documented in a number of publications, using either the comparison of randomized groups or pooling the data and investigating associations. There were some transient effects but, overall, there were no consistent indications of any effect of the dietary increase of the maternal n-3/n-6 LC-PUFA ratio on adipose tissue development or body composition until the age of 5 years [80]. Sophisticated ultrasound methods were used to determine fat development and distribution in the infants, which enabled a sex-specific description of the growth rates of different fat depots until the age of 5 years, again showing no intervention effect [81,82]. The intervention was found to affect the mRNA expression of certain genes in the placenta in a sex-specific way but did not influence maternal and cord blood leptin, which showed weak inverse associations with some parameters of infant growth [81,82,83]. Interestingly, for the first year of life, fat accumulation was found to be positively associated with the human milk supply of n-3 LC-PUFA, while ARA supply at 6 weeks was negatively associated with weight and body mass index (BMI) at age 6 weeks and 4 months, but no longer at age 12 months [79].

Thus, the INFAT study suggests that the pre- and postnatal increase of n-3 LC-PUFA does not affect the weight gain of the infants in a relevant way, which is in line with the conclusions of current reviews and meta-analyses. Randomized clinical trials do not provide conclusive evidence that n-3 LC-PUFA supplementation during pregnancy could lower the obesity risk of the offspring [80,84].

An impressive number of further randomized clinical trials have studied various doses of n-3 LC-PUFA supplementation during pregnancy or early postnatal life with respect to offspring anthropometry and/or on measures of adiposity, and the findings have been summarized in a number of systematic reviews and meta-analyses.

Li et al., in their meta-analysis, found significant positive effects of n-3 supplementation on birth weight and the length of gestation, but eight included trials following growth from the postnatal period up to an age of 7 years did not show a significant effect on weight or BMI [85]. A meta-analysis from 2014 included 6 RCTs with 2847 subjects and did not indicate any BMI effect from maternal n-3 supplementation, either for preschool or for school-aged children. In conclusion, there is no evidence that n-3 LC-PUFA supplementation during pregnancy and/or lactation would favorably affect child adiposity [86]. The review by Vahdaninia et al. considered weight, length, BMI, the sum of skinfolds, and percentage of body fat as outcome markers, which were recorded after randomized n-3 supplementation early in life and after follow-up periods between 6 months and 19 years [87]. They also concluded that the effects of n-3 LC-PUFA supplementation during pregnancy on obesity prevention remain unclear [87]. A review from 2021, including 27 RCTs with n-3 supplementation studies in healthy women [76], found that DHA/EPA supplementation doses of > 650 mg/day induced slightly higher birth weight and BMI and BMI z-score at 5–10 years (standardized mean difference 0.11, 95% CI 0.04–0.18), while there were no significant effects at smaller dosages [76]. The systematic reviews agree that n-3 supplementation during pregnancy increases pregnancy duration and reduces the risk of preterm birth [85,88,89,90], an effect potentially mediated by n-3 LC-PUFA-derived anti-inflammatory eicosanoids, while no lasting effects on obesity risks were established.

In non-breastfed infants, the effect of early postnatal LC-PUFA status on growth was studied in randomized clinical trials allocating infants to formulas with and without LC-PUFA. In an individual patient data-based meta-analysis, Rosenfeld et al. observed no association of LC-PUFA intake with weight until the age of 18 months [91]. Additionally, the long-term follow-up of intervention studies until 9 years of age did not detect differences in growth and cardiovascular risk markers between infants receiving formula with or without LC-PUFA [92]. These study results agree with the conclusion of a recent Cochrane review identifying no significant growth differences until preschool age with the addition of both DHA and ARA, or DHA only, to infant formulas [90].

## 4. Conclusions

There is a biologically plausible case for the relevance of perinatal PUFA status for later anthropometric development and obesity risk. The proposed mechanisms and observed relationships are complex, and the question remains open whether lower n-6 LC-PUFA or higher n-3 LC-PUFA levels are of more relevance, and whether the long-term effects may differ with different offspring ages [93].

Perinatal LC-PUFA status can be influenced by dietary modifications or supplementation. Several mechanisms have been described that could mediate LC-PUFA effects on the later development of obesity. However, available data in humans suggest that the influence of achievable modifications of the perinatal n-3/n-6 status is not sufficient to influence offspring obesity risk in the general population. Randomized intervention trials were performed in different countries with variable n-3 LC-PUFA intake, as evidenced by different DHA contents in human milk in the corresponding countries [25,94]. The outcome does not seem to depend on the habitual diet, but dose-response studies using the longer-term growth of the infants are still to be produced. Besides the general limitations of randomized intervention trials in nutrition [95], one must take into account that, so far, none of the FA supplementation trials included a pre-pregnancy period, so conclusions are limited to LC-PUFA effects during the second half of pregnancy and the postnatal part of the first 1000 days.

Considering the large body of evidence from mechanistic studies that support the potential of LC-PUFA to influence later obesity and the important need for effective obesity prevention, further studies seem justified to clarify the reasons why the results of model experiments could not be extrapolated to humans. This may include some less considered points, such as the LC-PUFA source, since LC-PUFA bioavailability might be different between fish oil, krill oil, microalgae and other products [96,97]. Furthermore, the quality of the dietary source of LC-PUFA, which is primarily reflected in the content of (per)oxidized FAs and organic pollutants, such as polychlorinated biphenyls or organochlorine pesticides, could be of importance [98,99]. As high contents of contaminants might suppress the beneficial effects, corresponding data should be reported for future trials. Even if there is no correlation between perinatal LC-PUFA status and obesity risk in the general population, there may be subgroups and settings where dietary modification of PUFA and LC-PUFA status may modulate obesity risk [100,101].
